# Supplementary material for: Exploring General Practitioners’ Knowledge, Attitudes, and Practices towards E-Cigarette Use/Vaping in Children and Adolescents: A Pilot Cross-Sectional Study in Sydney
Source: Int J Environ Res Public Health. 2024 Sep 16;21(9):1215. doi: 10.3390/ijerph21091215 (PMC11444199; doi:10.3390/ijerph21091215)
Supplement: Supplementary file 1 [file ijerph-21-01215-s001.zip › ijerph-3131477-supplementary.pdf]

Supplementary material S1

Participant Invitation Email

Dear Colleague,

This email is an invitation to participate in the study titled: "**Knowledge, attitude, and practice of general practitioners regarding the use of e-cigarettes in children more than 8 years of age and adolescents"**. This study is being conducted by the Department of Paediatrics at Nepean Hospital in partnership with the Prevention Education and Research Unit, WSLHD and the Department of Adolescent Medicine, The Children's Hospital at Westmead. You will be asked to complete an online questionnaire that explores the understanding, beliefs, and perceptions of the general practitioners working in NSW regarding the use of e-cigarettes in children and adolescents. Results from this study are expected to provide insight into addressing and managing this important issue in the vulnerable population group of children and adolescents.

It should take approximately 10–15 minutes to complete the online survey via the Quality Auditing Reporting System (QARS) survey platform. **Please participate in the survey only if you are a registered general practitioner or a registrar with the RACGP and are currently practising in NSW.** All data generated from the study will be held within Nepean Blue Mountains Local Health District (NBMLHD). The link to the survey can be found at the end of this invitation. All participation is voluntary and will be greatly appreciated. At the end of the online survey, you will be provided with a link to a free interactive educational tool for professionals working with young people who smoke.

This study has been approved by the (NBMLHD) Low and Negligible Risk Committee. The survey will require you to provide basic details about your professional practice but will not collect any personal or identifying information. The results may be published in a peer-reviewed journal or presented at conferences and will be completely anonymous.

Please do not hesitate to contact Dr. Habib Bhurawala if you have any questions or queries about the survey via the email address provided below.

Please feel free to contact the NBMLHD HREC Executive Officer via NBMLHD-Ethics@health.nsw.gov.au or 02 4734 1998 should you have any complaints, or questions about the study or being a study participant.

Thank you for your time and help.

Yours sincerely,

Dr Habib Bhurawala
Coordinating Principal Investigator
Staff Specialist Paediatrician
Nepean Hospital
E: habib.bhurawala@health.nsw.gov.au

Dr Rajiv Singh
Co-investigator
Paediatric Registrar
Nepean Hospital
E: Rajiv.Singh2@health.nsw.gov.au

Supplementary material S2

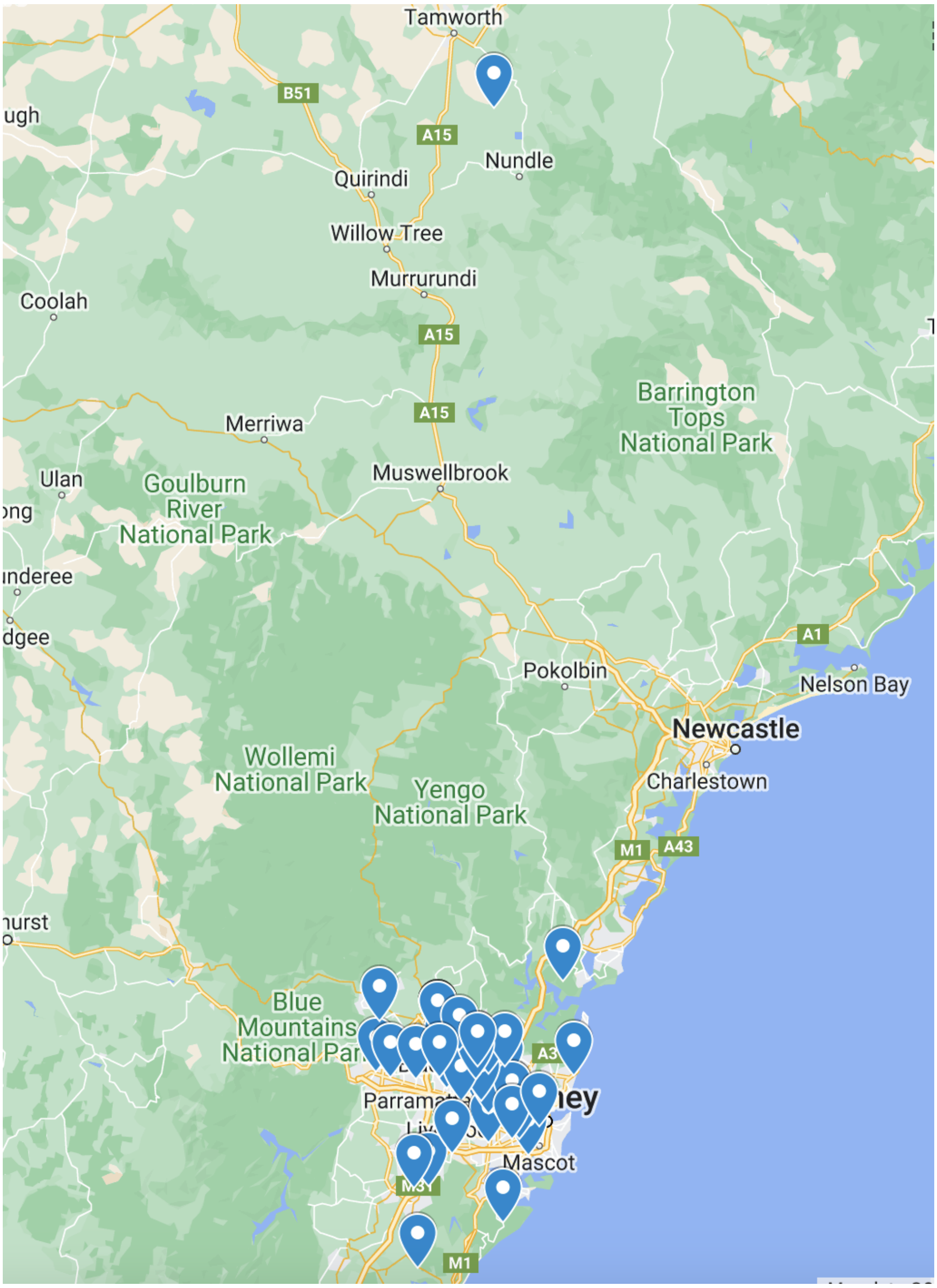

Figure S1. Map of the practice areas of GPs in New South Wales, Australia.
